# P14 The public’s understanding of antimicrobial resistance and its effect on flu vaccine uptake

**DOI:** 10.1093/jacamr/dlaf118.021

**Published:** 2025-07-14

**Authors:** Nicholas Hobbs, Lotte Wilde, Isabella Parker, Yana Khatri

**Affiliations:** University of Plymouth, Plymouth, UK; University of Plymouth, Plymouth, UK; University of Plymouth, Plymouth, UK; University of Plymouth, Plymouth, UK

## Abstract

**Objectives:**

To investigate the public’s understanding of antimicrobial resistance (AMR) and whether education could influence flu vaccine uptake, thereby contributing to the reduction of AMR.

**Methods:**

110 patients were surveyed from 5 pharmacies across Plymouth over 2 weeks in December 2024 and January 2025, to identify prior knowledge of AMR and intentions on receiving the flu vaccine. Patients were surveyed before and after watching a video, created with the assistance of AI, which explained the significance of AMR and the relationship between flu vaccine uptake and reducing inappropriate antibiotic use. The data gathered was statistically analysed to investigate changes in vaccine uptake and knowledge of AMR before and after the intervention.

**Results:**

We found that 40.9% of the population were not planning on getting the vaccine before our intervention, with the majority explaining this was due to concerns around side effects and beliefs that the flu vaccine is ineffective. After watching the video, only 10.2% stated that they were not inclined to receive a flu vaccine, a reduction of 30.7%. When asked to select the correct causes of AMR, 24.2% incorrectly identified vaccines as a cause or were ‘not sure’. After the video, only 6.3% were incorrect or ‘not sure’. When questioned about the links between AMR and flu vaccines, 43.8% of the responses were incorrect or not sure, which reduced to 30.4% after viewing the video. These findings support our aim and demonstrate statistically significant improvements in knowledge and vaccine intentions.

**Conclusions:**

A variety of factors influence the public's perception of AMR, highlighting the need for ongoing education. Our findings revealed a lack of public understanding of AMR, despite its growing importance. Although our sample size was limited, we demonstrated that targeted educational interventions could improve understanding of the link between flu vaccine uptake and AMR reduction, leading to increased vaccine intentions. Further educational interventions with patient populations on AMR could have a positive impact on attitudes both to flu vaccines and appropriate antibiotic use. Further studies are required to understand the role of patient and professional education into the reduction of AMR.

